# Utility of routine X-ray surveillance following hip sonography for developmental dysplasia in children: a single-center study spanning a decade

**DOI:** 10.1007/s00402-024-05695-7

**Published:** 2024-12-16

**Authors:** Matthias Wolf, Leon Haas, Stefanos Tsitlakidis, Julian Deisenhofer, Axel Horsch, Sébastien Hagmann, Katharina Susanne Gather

**Affiliations:** 1 Klinik für Orthopädie, Universitätsklinikum Heidelberg, Schlierbacher Landstraße 200a, 69118 Heidelberg, Germany; 2 Deutsches Gelenkzentrum Heidelberg, ATOS Klinik Heidelberg, Bismarckstr. 9-15, 69115 Heidelberg, Germany

**Keywords:** Developmental dysplasia of the hip (DDH), Radiography, Musculoskeletal sonography, Congenital hip dislocation, Orthopaedic procedure, Diagnostic imaging

## Abstract

**Introduction:**

This study evaluates the necessity of routine X-ray follow-ups in children with developmental dysplasia of the hip (DDH), identified through Graf hip ultrasound, a standard component of screening in Germany. The purpose of this study was to investigate the occurrence of radiological deterioration in hips that were initially diagnosed and treated according to established guidelines within a university-based risk-enriched cohort and to identify associated risk factors.

**Materials and methods:**

Patients diagnosed with developmental DDH from 2009 to 2018 with sonographically healthy hips (alpha > 64°) post conservative therapy and at least one follow-up X-ray by the age of two were analysed. Patients with significant comorbidities, syndromes, malformations, non-compliance with treatment, or missing X-ray data were excluded. Descriptive analysis of sonography, X-ray, and patient records were followed by univariate analysis and subsequent multiple logistic regression, identifying risk factors for severe and extreme dysplasia in X-rays.

**Results:**

Of the 450 included hips, 254 were classified as Graf Type 2a or higher, leading to treatment. Subsequent X-rays revealed severe dysplasia in 53 hips and extreme dysplasia in seven hips. Univariate analysis identified sex, initial Graf-Type, therapy type and duration as significantly associated with pathological radiographs. A regression model identified the initial Graf type as the predominant predictor with hip types 3a and 4, cast therapy and overhead extension as independent predictors.

**Conclusions:**

The data demonstrate pathological findings even after successful conservative treatment of DDH. Worsening of X-ray findings appear less frequent in mild dysplasia. These insights support routine radiographic follow-up assessments after successful conservative therapy. However, further dedicated studies are needed to determine whether patients with initially normal radiographs require radiographic follow-up.

## Introduction

Developmental dysplasia of the hip (DDH) is a common congenital skeletal deformity characterised by inadequate maturation of both the acetabulum and femoral head in infants, with an incidence of 2–4% in the population [22]. If left untreated or undiagnosed, DDH is a major risk factor for early-onset hip osteoarthritis in young individuals. The correlation between the severity of dysplasia and the development of osteoarthritis is well-documented [10, 12], highlighting the critical need for early detection and appropriate intervention.

The plasticity of the infant hip is greatest within the first 6 to 12 weeks of life [19], making early diagnosis and prompt treatment particularly effective. Since 1996, Graf’s ultrasound screening for DDH has been an integral part of a standardized preventive examination plan in Germany (U3, 4th-5th week of life), significantly reducing the incidence of persistent DDH [1, 21, 22]. Treatment varies depending on the severity of the condition but aims to harness the hip’s natural maturation potential.

Despite successful conservative treatment in infancy, several follow-up radiological controls are recommended for these patients up to skeletal maturity [15]. These controls aim to detect recurrences or late onset dysplasia of the contralateral side. However, the necessity of such routine X-ray controls, especially in patients with initially mild dysplasia, has been questioned due to concerns over radiation exposure and potential psychological impact on both parents and patients.

We conducted a retrospective analysis of all patients diagnosed with DDH in the first weeks of life at our institution from 2009 to 2018, who had undergone successful treatment and showed sonographically healthy hips (alpha angle > 64°) post conservative therapy. Through descriptive, univariate analysis and multiple logistic regression, we aim to understand the incidence of and predictive factors for radiological deterioration (severe or extreme dysplasia) and to assess the efficacy of routine radiological monitoring as a surrogate parameter for preventing late complications.

This investigation could assist in refining future guidelines on the management of DDH, particularly concerning the frequency and necessity of radiological follow-up. It aims to balance the risks of over-exposure to diagnostic radiation against those of underdiagnosis.

## Materials and methods

A retrospective clinical analysis was conducted on patients with DDH at our university hospital from 2009 to 2018 who initially presented in infancy. Suspicious findings from paediatric ultrasounds at recommended screening check-ups (U3, recommended at 4–5 weeks, required within 3–8 weeks of age in Germany) prompted further evaluation and referral. Some patients were referred even earlier due to family history or suspicious findings upon discharge from the hospital after birth. Subsequent hip ultrasounds were performed at our institution to confirm the initial diagnoses. Guideline-based treatment was conducted for confirmed DDH cases. Routine pelvic X-ray follow-ups were suggested as outlined by German guidelines, extending into adolescence, to monitor for recurrences or contralateral onset. Clinical records provided ultrasound, treatment, and follow-up data. Pelvic X-rays were analysed using TraumaCad® (Version 2.5, Brainlab AG, Munich, Germany) for DDH monitoring. Examples of sonographic and X-ray measurements are provided in Appendix fig. 6.

The inclusion criteria were children diagnosed with DDH (2009–2018) within the first weeks of life, treated with reposition, retention and maturing according to German guidelines for DDH [15], with sonographically healthy hips post-treatment (alpha angle of greater than 64 degrees) and at least one pelvic X-ray available before two years of age. Exclusion criteria included known syndromes or suspected neurological origins of DDH, severe comorbidities, non-compliance with treatment guidelines, insufficient treatment documentation, and lack of follow-up X-rays.

Patient and treatment details, along with associated risk factors, were documented. Descriptive statistics examined hip side, gender, risk factors, and initial sonographic hip types. Therapy durations, complications, and surgical interventions were analysed. The primary endpoint was the presence of severe or extreme dysplasia according to Tönnis in initial pelvic X-rays, see Appendix Table 4 [17, 18]. Subsequent X-ray controls formed secondary endpoints, analysing all available data. Postoperative X-rays were not included in cases where surgical correction was necessary. Statistical significance was set at 5% (p ≤ 0.05), using SPSS® V 29 (Armonk, NY, USA).

Following descriptive analysis, univariate analysis assessed associations among parameters. Based on these results, multiple logistic regression models were developed to test for independent predictive capabilities.

## Results

**Fig. 1:**
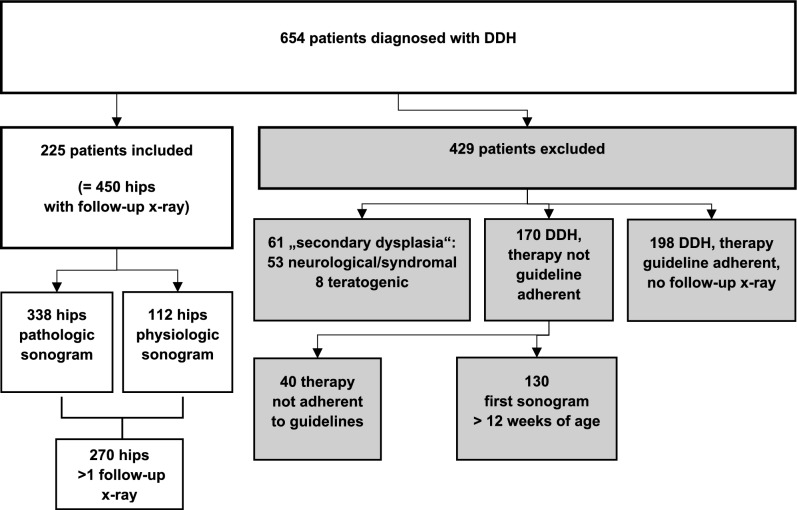
Flowchart of study participants—inclusion and exclusion of patients according to the respective criteria. DDH-developmental dysplasia of the hip, all patients were referred due to suspected DDH based on sonographic findings or risk factors. This report includes only patients with sonographically confirmed DDH

A retrospective analysis of 654 patients revealed 225 patients (450 hips) with DDH meeting the inclusion criteria. Among the excluded 429 patients, 61 had secondary hip dysplasia, primarily of neurological or syndromic origin, while eight had teratogenic dysplasia. The follow-up data of 368 patients was incomplete, mostly due to therapy non-compliance or discontinuation and consultations for second opinions on external treatment. Of these, 170 patients did not receive guideline adherent treatment, mostly due to late referral or presentation after 12 weeks of age. Despite recommendation 198 patients did not receive follow-up x-rays (see Fig. 1). The high number of single consultations or non-guideline-compliant therapies is due to the centre’s position as a maximum care provider. Patients are frequently presented for second opinions and continue treatment locally, while other patients continue treatment at our centre that has been initiated elsewhere. All these patients were excluded for cohort homogeneity.

Of the 225 patients included, 338 (75%) hips were identified as abnormal on ultrasound. Expectedly, 88.4% (199/225) of the patients were female, with dysplasia affecting both hips in 79.2% (268/338) and the left hip in 15.3% (52/338). Risk factors included breech position 20.4% (46/225 patients) and positive family history in 30.2% (68/225 patients).

**Fig. 2:**
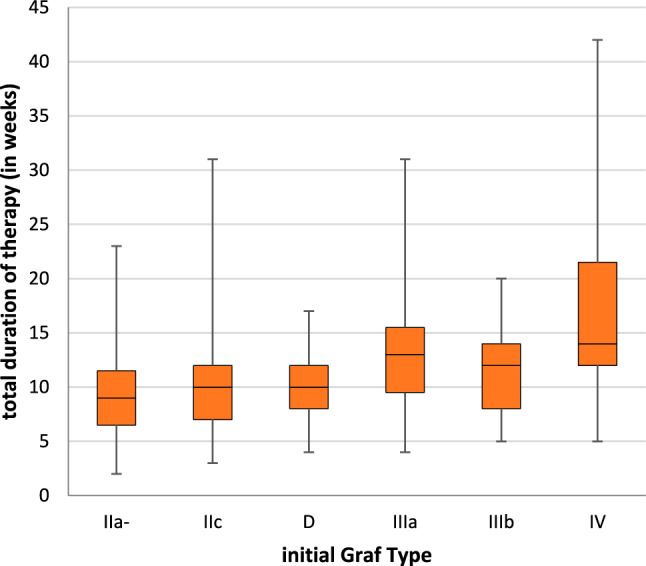
Initial Graf types and their total duration of therapy (in weeks)

In accordance with the inclusion criteria, all patients received guideline-compliant therapy. Among the study group, 254 hips exhibited Graf Type 2a or higher. While double-diapering was not considered therapy, overhead extension, spica casting and orthosis (Tübinger, Pavlik, etc.) were used sequentially depending on Graf-type in compliance with the guidelines. Therapy continued until an alpha angle of greater than 64° was achieved. Therapy duration is illustrated in Fig. 2 and Appendix Table 5. Additionally, 270 hips had further radiographic follow-ups after 2 years.

**Fig. 3:**
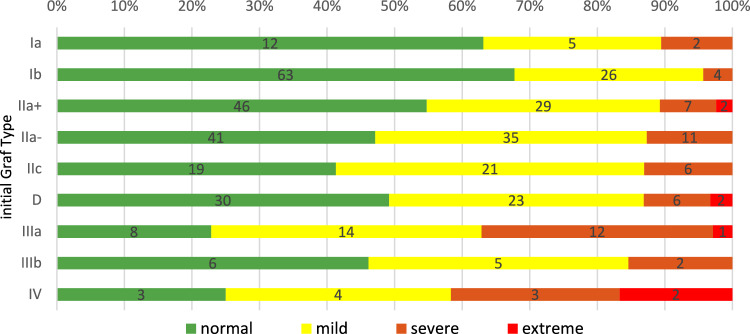
Grade of dysplasia of the initial x-ray by initial Graf type

Follow-up radiographs revealed severe dysplasia in 53 hips and extreme dysplasia in seven hips. Notably, pathological radiographic findings were observed even in cases initially classified as mild sonographic dysplasia according to Graf (see Table 1 and Fig. 3).

**Table 1:** Initial grade of dysplasia by initial Graf type

	Grade of dysplasia of the first x-ray				Total (100%)
	Normal	Mild	Severe	Extreme	
Initial Graf type					
Ia	12 (63.2%)	5 (26.3%)	2 (10.5%)	0	19
Ib	63 (67.8%)	26 (27.9%)	4 (4.3%)	0	93
IIa +	46 (54.7%)	29 (34.5%)	7 (8.3%)	2 (2.3%)	84
IIa−	41 (47.1%)	35 (40.2%)	11 (12.6%)	0	87
IIc	19 (41.3%)	21 (45.6%)	6 (13%)	0	46
D	30 (49.1%)	23 (37.7%)	6 (9.8%)	2 (3.2%)	61
IIIa	8 (22.8%)	14 (40%)	12 (34.2%)	1 (2.8%)	35
IIIb	6 (46.1%)	5 (38.5%)	2 (15.4%)	0	13
IV	3 (25%)	4 (33.3%)	3 (25%)	2 (16.6%)	12
Total	228	162	53	7	450

Mean age at initial x-ray 16.2 months (± 3.1, range 8–31)

Univariate analysis showed significant associations between pathological radiographic findings and sex (p = 0.032), overhead extension (p < 0.001), cast therapy (p = 0.003), Tübinger orthosis (p = 0.015), sonographic hip type (p < 0.001) and therapy duration (p = 0.004) for pathological radiographic findings. Breech presentation (p = 0.07) and family history (p = 0.762) did not show significance, likely due to the referral bias towards cases of suspected hip dysplasia. For full results, see Table 2.

**Table 2:** Univariate analysis of the association with pathological radiographic findings

	Χ^2^	Asymptotic significance (2-sided)
Family history^1^	0.092	0.762
Breech position^1^	3.280	0.070
Hip side^1^	2.769	0.096
Sex^1^	4.579	0 0.032*
Overhead extension^1^	13.282	< 0.001*
Cast therapy^1^	9.030	0.003*
Tübinger orthosis^1^	5.880	0.015*
Initial Graf type^1^	32.260	< 0.001*
Therapy duration (in weeks)^2^		0.004*

^1^Pearson’s chi-squared test

^2^Nonparametric Mann–Whitney-U Test

*indicates p < 0.05

The significant parameters were subsequently analysed using multivariate logistic regression models. Ultimately, Graf type emerged as the predominant predictor, with hips classified as type 3A (p < 0.001; OR = 10.44) and type 4 (p < 0.001; OR = 12.62) identified as independent predictors. Additional independent predictors of pathological X-ray findings included cast therapy (p = 0.045, OR 4.93) and overhead extension (p = 0.012, OR = 11.33). The other parameters did not show independent associations. Therefore, the initial sonographic hip type (Graf type) and therapy with overhead extension or cast were identified as independent predictors. Given that the therapy mode is inherently linked to the Graf type, overhead extension or cast therapy reflect higher initial sonographic Graf types. In summary the data suggest that initial disease severity correlates with the likelihood of pathological findings on follow-up X-rays.

**Fig. 4:**
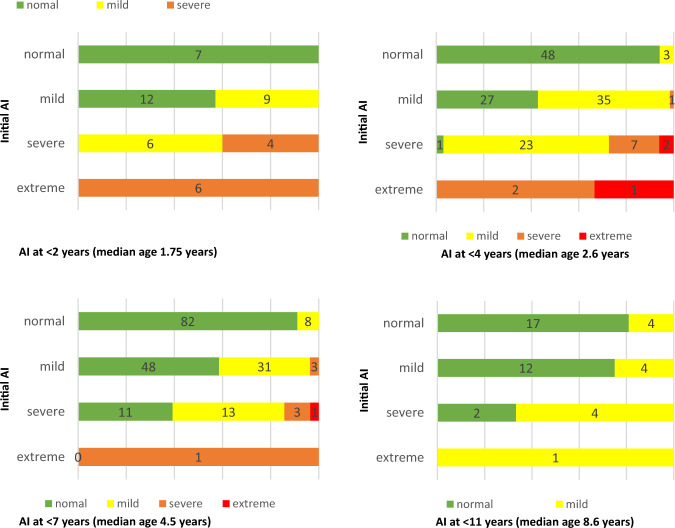
Comparison of Acetabular Index at timepoints 1 through 4 to the initial radiograph

**Fig. 5:**
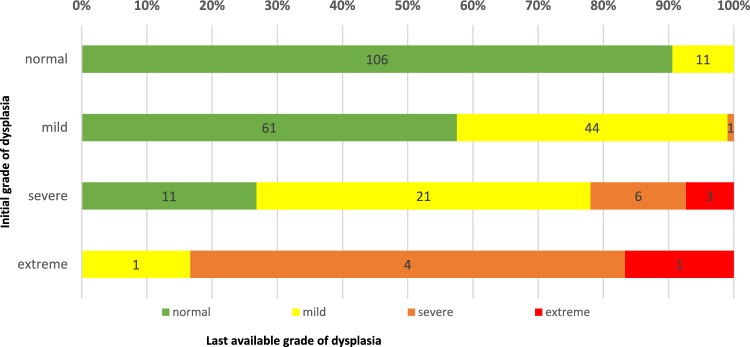
Initial grade of dysplasia by last available grade of dysplasia

Extended follow-up data were available for 135 patients after a mean of 55, 65 months (± 26.51, range 17 to 133). The additional radiographs were categorised as follows: the first radiograph was defined as up to the second year of life, the second radiograph up to the fourth year of life, the third up to the eighth year of life, and the fourth up to adulthood. There was a general tendency for improvement in the acetabular index (AI) during radiological follow-up. Notably, patients with initially normal findings did not require therapy throughout the entire extended follow-up period. For more detailed information, refer to Table 3 and Figs. 4 and 5. When comparing the radiographs taken at these respective time points with the initial radiograph, significant deterioration was observed in 4 hips at the second and third radiographs, with progressing from mild to severe dysplasia according to the acetabular index. An example case is shown in Appendix fig. 7.

**Table 3:** Initial grade of dysplasia by last available grade of dysplasia

		Last available grade of dysplasia					Lost to follow-up
		Normal	Mild	Severe	Extreme	Total	
Initial grade of dysplasia							
Normal	106 (90,5%)		11 (9,5%)	0	0	117 (100%)	111
Mild	61 (57,5%)		44 (41,5%)	1 (0,9%)	0	106 (100%)	56
Severe	11 (26,8%)		21 (51,2%)	6 (14,6%)	3 (7,3%)	41 (100%)	12
Extreme	0		1 (16,7%)	4 (66,7%)	1 (16,7%)	6 (100%)	1
Total	178		77	11	4	270	180

Initial AI-grade by last available AI-grade, showing changes from normal, mild, severe, to extreme categories with total numbers, rates and those lost to follow-up

Eight hips underwent surgical correction. One additional patient did not follow-up after surgery was indicated. The timing of surgical indications was distributed as follows: one based on the initial radiograph, one after the second radiograph, four after the third radiograph, and five after the fourth radiograph. All surgeries involved classical derotation varisation osteotomy (DVO) and Dega acetabuloplasty in eight cases. For more detailed information, see Appendix Table 6.

## Discussion

Screening for developmental dysplasia of the hip (DDH) has been a mandatory component of preventive paediatric examinations in Germany since 1996, emphasised by the guidelines of the German Society for Orthopaedics and Orthopaedic Surgery [15]. They recommend referral to a specialised centre upon sonographic suspicion of DDH [15], with the aim to shorten diagnosis and hasten the initiation of appropriate therapy [15]. High-quality standards for ultrasound examinations and the use of the Graf classification are mandated [15]. In contrast, the protocols in the United States and the United Kingdom favour selective ultrasound post initial clinical examination, reserved for cases of clinical concern or presence of risk factors [11, 16]. This approach has increased the diagnosis rates of DDH without reducing the necessity for surgical interventions in the UK [11]. In Germany, universal screening has been successful in reducing the number of surgeries [1, 5, 21].

Our study cohort had a notable prevalence of risk factors: breech position (20%), family history (30%) and a predominantly female demographic (88.4%). Previous studies have highlight similar observations, with a meta-analysis reporting breech position prevalence between 1.1% and 6.5% [2] with associated odds ratios of 5.7 for breech position, 4.8 for family history and 3.8 for female demographic [2]. Recent studies support these findings [4, 9]. The prevalence of risk factors in this study is likely attributed to the pre-selection of patients with DDH patients via screening, which we are not primarily involved in.

The mean therapy duration in German centres ranges from 7.3 to 16.1 weeks [6, 7, 14]. These data focused on patients with high Graf-types and early treatment initiation. In our study the mean treatment duration was 11.36 weeks, extending significantly for those classified as Graf Type IV, consistent with previous studies [6, 7, 14].

The primary treatment objective is the development of a mature hip indicated by an alpha angle of > 64°, reflecting a healthy and well centred hip, expected to develop normally after walking onset. Particularly for initially low-grade dysplasia, the development of late dysplasia is not anticipated. Nevertheless, the German guidelines recommend pelvic x-rays at the age of two, pre-school age, pre-puberty, and post growth completion [15]. Communicating these recommendations to parents can be delicate, despite evidence showing a very low risk associated with radiation exposure [20]. A systematic review and meta-analysis revealed occurrence of late dysplasia despite universal screening with a negative trend observed in societies with universal screening like Germany, although this did not reach statistical significance [8].

A study from the United States found abnormal radiographs by six months of age in 17% of cases following successful treatment of DDH, and in 33% by one year of age, using a comparable approach to our study [13]. Similarly, our longitudinal follow-up indicated 18.4% (60) of successfully treated hips exhibited pathological radiographic findings, with severe or extreme dysplasia up to two years of age. The data show evidence of both late-onset dysplasia and recurrent dysplasia. Even sonographically healthy hips and hips with mild dysplasia showed worsening over time, with one case of mild dysplasia eventually even requiring surgical correction. In the extended follow-up, patients with initially normal pelvic X-rays did not experience treatment-requiring deterioration, although pathological radiographic findings did appear in patients with initially mild dysplasia.

Kubo et al. reported on 116 patients with 161 unstable hips treated off-label with the Tübingen splint [7]. A notable aspect was the early initiation of therapy; if a stable hip was not achieved by the sixth week of life, treatment was switched to a spica cast [7]. The applied therapy, methodology and cohort allow for direct comparison of the results [7]. Of the 75 hips included in their study, initial radiographs showed 31 (41.3%) normal, 31 (40.3%) mildly dysplastic, and 13 (17.4%) severely dysplastic hips [7]. By the second radiograph (under 4 years), 53 (70.7%) were normal, 19 (25.3%) mildly dysplastic, and 3 (4.0%) severely dysplastic [7]. The third radiograph (under 8 years) showed 60 (80%) normal, 14 (18.7%) mildly dysplastic, and 1 (1.3%) severely dysplastic hip [7]. Six hips (8.0%) worsened during this time, with five transitioning from normal to mild dysplasia and one from mild to severe dysplasia [7].

In a subsequent study from 2023, an expanded cohort of 38 hips was examined including the fourth radiograph and following the same inclusion criteria [6]. The follow-up times averaged 13.7 months, 32.3 months, 69.2 months, and 118.7 months for the first through fourth radiographs, respectively [6]. The study reported on the AI for the first three radiographs, with the initial radiograph showing 52.8% of hips as mature, 38.9% as mildly dysplastic, and 8.3% as severely dysplastic [6]. Approximately 78% of cases showed normal findings in the second and third radiographs, with 22% showing mild dysplasia [6]. From the third to the fourth radiograph, a slight deterioration was noted in the centre-edge (CE) angle, from 16.7% mild dysplasia in the third to 18.9% in the fourth radiograph [6]. The authors highlight that the most significant changes occurred between the first and second radiographs, with only isolated instances of worsening or improvement thereafter [6]. Despite the observed progression in some cases, the authors advocate for radiographic follow-ups due to the minimal radiation exposure and the benefits of monitoring these changes [6].

Our cohort data align with these findings [6, 7]. Most hips in our cohort showed abnormalities by the second radiograph, already indicating half of the surgical procedures. The third radiograph in our cohort was obtained earlier than in Kubo et al.’s study (mean 54 months vs. 69 months). While the sample size of Kubo’s long-term follow-up study is small [6], their long-term outcomes are consistent with our findings. Of the 44 available fourth radiographs, three showed marginally dysplastic AI but also CE angles within normal range, resulting in no surgical consequences.

Thus, the data from this study reveal pathological findings on initial pelvic X-rays in successfully treated DDH. These findings seem to improve on the second X-ray in many cases, which is consistent with the experience from other studies [3]. Even though only five patients (9 hips) in this pre-selected cohort required surgery, diagnosing them remains essential. Also, deterioration of initially normal hips on ultrasound to dysplasia on radiographs supports continued radiographic follow-up in unilateral DDH patients to monitor the contralateral hip.

In summary, the data show a significant association between Graf-Type (independently 3a and 4) and the type and duration of therapy with pathological X-ray findings. This primarily suggests that initial severity of the disease correlates with the likelihood of pathological X-rays. Consequently, routine X-ray follow-ups are recommended for patients with initially severe disease. Patients with initially low Graf types also showed pathological findings during follow-up, with isolated cases requiring surgery. Therefore, the benefits of detecting late dysplasia appear to outweigh the risks of X-ray follow-up in patients with an initial diagnosis of DDH. German guidelines suggest follow-up X-rays before 2 years of age, before 5 years of age (pre-school), and before puberty. A final X-ray is suggested after reaching adulthood, although in clinical practice, this is rarely obtained at the initially treating facility.

Further studies may justify omitting additional radiographs for patients with initially normal AI and the pre-puberty radiograph for all patients if the absence of clinical consequences for these patients is confirmed. However, the sample sizes of both Kubo’s [6] and our study do not allow for generalisation of these findings.

### Limitations

This is a retrospective cohort study, limiting its ability to control for confounding factors. However, in the context of DDH given the mandatory screening a prospective design is not feasible. The study also suffers from selection bias. The sample consists of a high-risk cohort of patients referred to a specialised centre, which may not represent the general population. This design enables the detection of rare events like late dysplasia, due to its risk enriched environment and compares well to other centre driven studies. Due to the different healthcare systems and recommendations the results are mainly applicable to Germany. Follow-up Data are incomplete, as a significant portion of the initial patient cohort was excluded due to inadequate follow-up, therapy non-compliance or discontinuation and patients who sought external treatment. This diagnostic gap could limit the interpretation of the results.

## Conclusion

To our knowledge, this study represents the first longitudinal follow-up of radiographic images following successful therapy after universal ultrasound screening for DDH. The data reveal pathological findings even after successful conservative treatment of DDH. However, worsening of X-ray findings appears to be less frequent in cases of mild dysplasia. Reflecting disease severity, Graf-type, therapy type and duration were significantly associated with radiological worsening. The insights gained from this study support the continued recommendation for routine radiographic follow-up after successful conservative therapy. However, further studies are needed to determine whether patients with initially normal radiographic findings require ongoing radiographic follow-up.

## Figures and Tables

**Fig. 6:**
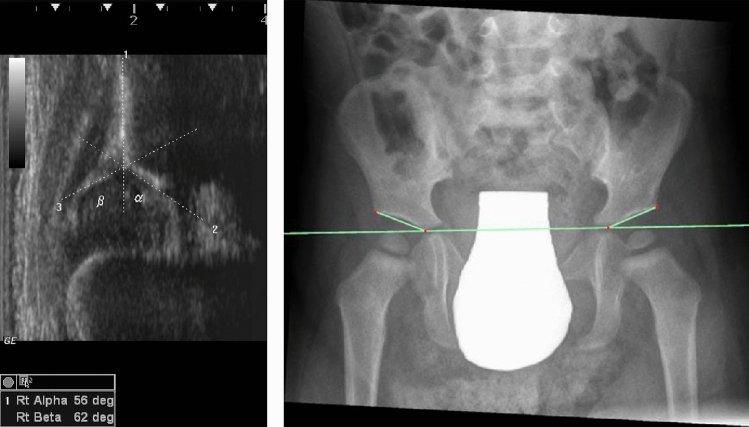
Examples of sonographic and X-ray measurements. Left image: right hip sonogram, 5-week-old male, Graf-Type 2a plus, initiating sonographic follow-up. Right image: pelvic X-ray, 2-year-old female, AI of 22° on both sides, routine follow-up

**Fig. 7:**
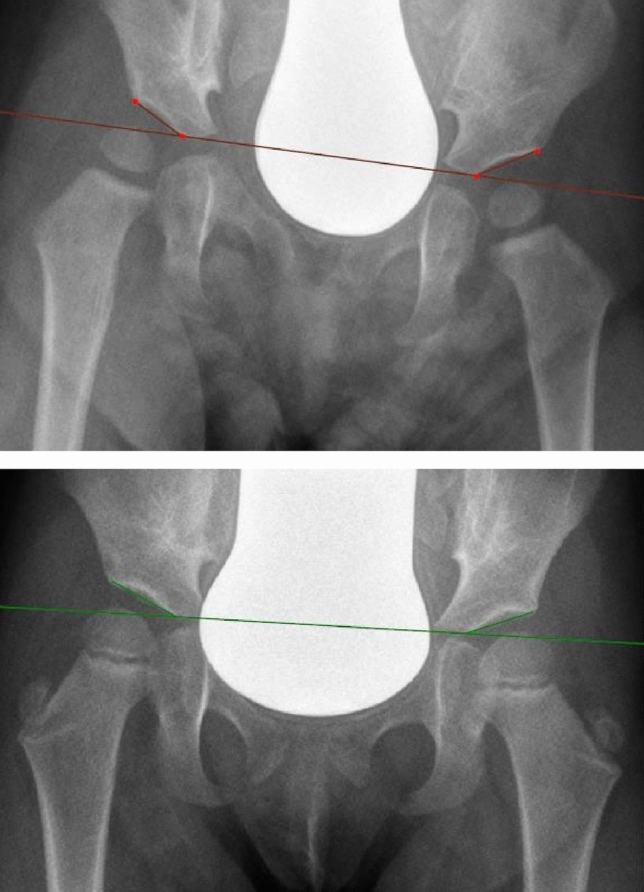
Example case of AI-deterioration. Pelvic X-ray at 17 months, AI left 30° (severe dysplasia), AI right 29° (mild dysplasia). Pelvic X-ray of the same patient at 45 months, AI left 21° (mild dysplasia), AI right 26° (severe dysplasia). The left hip improved, the right hip deteriorated from mild to severe dysplasia in the age-adjusted measurement (see Appendix Table 4)

## Data Availability

The data that support the findings of this study are not openly available due to reasons of sensitivity and are available from the corresponding author upon reasonable request.

## Appendix

See Figs. 6, 7 and Tables 4, 5, 6

**Table 4:** Normal values and degrees of deviation of the Acetabular Index (grade of dysplasia) classification system of the task force for hip dysplasia of the DGOT. (Tönnis, 1985)

Age	Mean AI	Grade 1 (normal)	Grade 2 (mild)	Grade 3 (severe)	Grade 4 (extreme)
5. month–2. year	20	< 25	25 to < 30	30 to ≤ 35	> 35
2.–3. year	18	< 23	23 to < 28	28 to ≤ 33	> 33
3.–7. year	15	< 20	20 to < 25	25 to ≤ 30	> 30
7.–14. year	10	< 15	15 to < 20	20 to ≤ 25	> 25

**Table 5:** Initial Graf types and their average duration of therapy

Graf type	(N) hips	Average duration of therapy in weeks (± SD)
IIa-	87	9.48 (± 4.531)
IIc	46	9.89 (± 5.271)
D	61	10.11 (± 3.362)
IIIa	35	13.54 (± 5.962)
IIIb	13	10.77 (± 3.876)
IV	12	16.58 (± 9.268)
Total	338	11.36 (± 5.404)

*N* number

**Table 6:** Descriptives of surgical procedures

Patient (number) and hip side	Initial Graf-type	Initial AI-Grade	last AI-grade (before surgery)	Age at surgery	Surgical procedure
1 left	IIa-	Severe	Extreme	55 months	DVO/Dega
1 right	Ib	Severe	Severe	55 months	DVO/Dega
2 left	IIc	Severe	Extreme	35 months	DVO/Dega
2 right	Ia	Severe	Extreme	35 months	DVO/Dega
3 left	IIa +	Extreme	Extreme	23 months	DVO
3 right	IIa +	Extreme	Severe	23 months	DVO
4 right	IV	Extreme	Severe	23 months	DVO/Dega
5 left	IV	Extreme	–	19 months	DVO/Dega

Descriptives of surgical procedures detailing patient number, hip side, initial Graf-type, initial AI-Grade, last AI-grade before surgery, age at surgery, and surgical procedure
